# Case Report: *De novo DDX3X* mutation caused intellectual disability in a female with skewed X-chromosome inactivation on the mutant allele

**DOI:** 10.3389/fgene.2022.999442

**Published:** 2022-10-10

**Authors:** Yixi Sun, Yangwen Qian, Hai-Xi Sun, Min Chen, Yuqin Luo, Xiaojing Xu, Kai Yan, Liya Wang, Junjie Hu, Minyue Dong

**Affiliations:** ^1^ Department of Reproductive Genetics, Women’s Hospital, School of Medicine, Zhejiang University, Hangzhou, Zhejiang, China; ^2^ Key Laboratory of Reproductive Genetics, Ministry of Education (Zhejiang University), Hangzhou, Zhejiang, China; ^3^ Key Laboratory of Women’s Reproductive Health of Zhejiang Province, Women’s Hospital, School of Medicine, Zhejiang University, Hangzhou, Zhejiang, China; ^4^ College of Life Sciences, University of Chinese Academy of Sciences, Beijing, China

**Keywords:** *DDX3X*, skewed X-chromosome inactivation (XCI), intellectual disability, escaping X-chromosome inactivation (XCI), RNA sequencing

## Abstract

Skewed XCI plays an important role in the phenotypic heterogeneities of many X-linked disorders, even involving in diseases caused by XCI-escaping genes. *DDX3X*-related intellectual disability is more common in females and less common in males, who usually inherit from unaffected heterozygous mothers. As an X inactivation (XCI) escaping gene, the role of skewed XCI in the phenotype of *DDX3X* mutant female is unknown. Here we reported a *DDX3X*: c.694_711dup18 *de novo* heterozygous mutation in a female with intellectual disability on the maternal X chromosome on the basis of SNPs detected by PCR-sanger sequencing. *AR* assay revealed that the maternal mutant X chromosome was extremely inactivated in the proband. Using RNA sequencing and whole-exome sequencing, we quantified allelic read counts and allele-specific expression, and confirmed that the mutant X chromosome was inactive. Further, we verified that the mutant *DDX3X* allele had a lower expression level by RNA sequencing and RT-PCR, and the normal and mutated *DDX3X* expression accounted for respectively 70% and 30% of total. In conclusion, we found a symptomatic female with extreme skewing XCI in the *DDX3X* mutant allele. It was discovered that XCI in the mutant allele was insufficient to reverse the phenotype of *DDX3X*-related neurodevelopmental disorder. It contributed to a better understanding of the role of skewed XCI in phenotypic differences, which can aid in the genetic counseling and prenatal diagnosis of disorders in females with *DDX3X* defects.

## Introduction

Mutations in X-linked genes account for approximately 5%–10% of the causes of intellectual disability (ID) in males, whereas females with X-linked ID have much lower penetrance and phenotypic severity than hemizygous males (Dobyns et al., 2004; Lubs et al., 2012; Dikow et al., 2017). Female carriers with X-linked variants are generally thought to be protected by functional mosaicism caused by random X chromosome inactivation (XCI) or skewed XCI that favors the wild-type allele (Cotton et al., 2015; Kellaris et al., 2018).

*DDX3X* maps to Xp11.3–11.23 and is an XCI-escaping gene (Lahn and Page, 1997; Cotton et al., 2015). It encodes an ATP-dependent “DEAD-box” RNA helicase involved in numerous cellular processes; it is essential for cortical development, which is required in neural progenitors to produce cortical neurons during development (Linder et al., 1989; Kellaris et al., 2018; Lennox et al., 2020; Mo et al., 2021). *DDX3X* variants are one of the most common causes of ID, accounting for 1%–3% of females with unexplained ID (Snijders Blok et al., 2015). Around 848 individuals have been identified (https://ddx3x.org/). In general, *DDX3X*-related ID is more common in females with *de novo* variants and less common in males, who usually inherited from unaffected heterozygous mothers except few cases of *de novo* male patients (Snijders Blok et al., 2015; Wang et al., 2018; Nicola et al., 2019; Tang et al., 2021). Moreover, there are phenotypic differences in females with *DDX3X* variants.

Skewed XCI plays an important role in the phenotypic heterogeneities of many X-linked disorders, even involving in diseases caused by XCI-escaping genes (Sun et al., 2019). Although XCI-escaping genes are expressed from the inactive X chromosome (Xi) in all cells, their expression levels relative to active X chromosome (Xa) levels are usually reduced (Carrel and Willard, 1999). As similar with most of XCI-escaping genes, transcripts of *DDX3X* are not fully expressed from the Xi, escaping ratios of *DDX3X* are ranging from 29% to 61% (Garieri et al., 2018). Expressions of wild-type and mutant *DDX3X* are affected by skewed XCI, so we tried to explore if skewed XCI might dictate the clinical phenotypes of females with *DDX3X* mutations, which could aid in genetic counseling for the prenatal diagnosis of *DDX3X*-related intellectual disability. Here we investigated an intellectual disability girl with a *de novo DDX3X* mutation. By PCR-sanger sequencing, we defined that the mutation occurred from which X chromosome, and further detected the methylation and transcription levels of the X chromosome by *AR* assay and RNA sequencing.

## Materials and methods

### Patient

The proband was a 10-year-old girl without family history. The symptoms of the proband included mild to moderate intellectual disability, delayed psychomotor development, poor speech development, infantile hypotonia, autism, and behavior problems. The patient was born after a normal pregnancy and delivery. She could not sit without support at 18 months. When seeing a doctor at the age of 19 months, her gross motor and fine motor skills were obviously delayed, her abilities of grasp, visual movement, stationary and locomotivity were respectively equivalent to 12, 13, 11, and 9 months of age. She began to walk at 2 years. At the age of 26 months, her language level was equivalent to the normal 12.7-month-old child (Sym score: 5). At the age of 7 years, she was diagnosed as autism spectrum disorder, the scores of childhood autism rating scale and autistic behavior checklist were 32 and 75. Spike and slow wave complex sporadically showed in video electroencephalogram (VEEG). Brain magnetic resonance imaging (MRI) was normal. By color doppler ultrasound, abnormal cerebral blood flows were detected in middle cerebral artery (left: +3SD, right: +3SD) and anterior cerebral artery (left: +3SD, right: +2-3SD) at 7 years.

### DNA/RNA extraction, PCR, RT-PCR, and T-A cloning

Genomic DNA and total RNA of peripheral blood mononuclear cells (PMBCs) were respectively extracted with the Gentra Puregene Kit (Qiagen, Germany) and RNAiso Plus (Takara, Japan). Extracted total RNA was reverse-transcribed using PrimeScript™ RT Master Mix (Takara, Japan). PCR was performed using GoldStar Best Master Mix (CWBIO, Beijing). Primer sequences were listed below. *DDX3X*-DNA-F: AGA​CTT​GAT​GGC​TTG​TGC; *DDX3X-*DNA-R: TTT​CTG​GCT​TCC​TCG​TAG; *DDX3X-*RNA-F: CAG​TGA​TGT​TGA​GAT​GGG​AGA​A; *DDX3X-*RNA-R: AAA​CCA​CGC​AAG​GAC​GAA. T-A cloning was performed by Hieff Clone™ Zero TOPO-TA Cloning Kit (Yeasen, Shanghai) according to the manufacturer’s instruction. Sanger sequencing were performed by ABI 3730 DNA analyser.

### X inactivation analysis

To analyze XCI in DNA methylation level, XCI in the family was analyzed using PCR amplification of the *AR* gene in blood, as previously described (Allen et al., 1992). Primers sequences were listed below. *AR-*F: TCC​AGA​ATC​TGT​TCC​AGA​GCG​TGC (labeled by 5′6-FAM); *AR-*R: GCT​GTG​AAG​GTT​GCT​GTT​CCT​CAT. Amplified products were run on an ABI 3500 genetic analyzer.

### Whole exome sequencing and RNA sequencing

Whole-exome sequencing (WES) and RNA-sequencing were performed by Biomarker Technologies Corporation (Beijing, China). The sequencing libraries were generated using the NimbleGen SeqCap EZ Human Exome V3 (Roche, Basel, Swiss) and NEBNextR Ultra™ Directional RNA Library Prep Kit for Illumina (NEB, United States). Clustering of the index-coded samples was performed on a cBot Cluster Generation System (Illumina, United States). After cluster generation, the library preparations were sequenced on an Illumina HiSeq X Ten platform.

Burrows-Wheeler Aligner v0.7.13-r1126 was used to align each sample’s clean reads with the reference genome using default parameters. Alignment files were converted to BAM files using SAMtools software. Variant calling was performed for all samples by using the Haplotype Caller in GATK software.

To calculate the proportion of expressed wild type allele and mutant allele of *DDX3X* at the RNA level, we mapped the RNA-seq reads to a modified cDNA sequence of *DDX3X* gene (NM_001193416.2), in which the 18-bp wild type allele sequences on exon 8 were removed, using BWA (Version: 0.7.17) (Li and Durbin, 2009) with parameters “aln -t 12 -o 3 -e 35 -d 0 -i 0 -l 100 -k 0 -M 1 -O 1 -E 1 -N -n 0.0001.” Then an in-house Perl script was used to extract the wild type allele sequence or mutant allele sequence from the mapped reads.

### Allelic read counts and allele-specific expression

To distinguish the transcription of alleles on the two X chromosomes, we explored the exome sequencing data for heterozygous informative SNPs on the X chromosome in II.1 to assess the parental origin. Combining RNA sequencing, informative SNPs were quantified allelic read counts and allele-specific expression (ASE).

## Result

### *DDX3X*: c.694_711dup18 *de novo* heterozygous mutation was found in the proband using whole-exome sequencing and occurred on the maternal X chromosome detected by PCR-sanger sequencing

**FIGURE 1:**
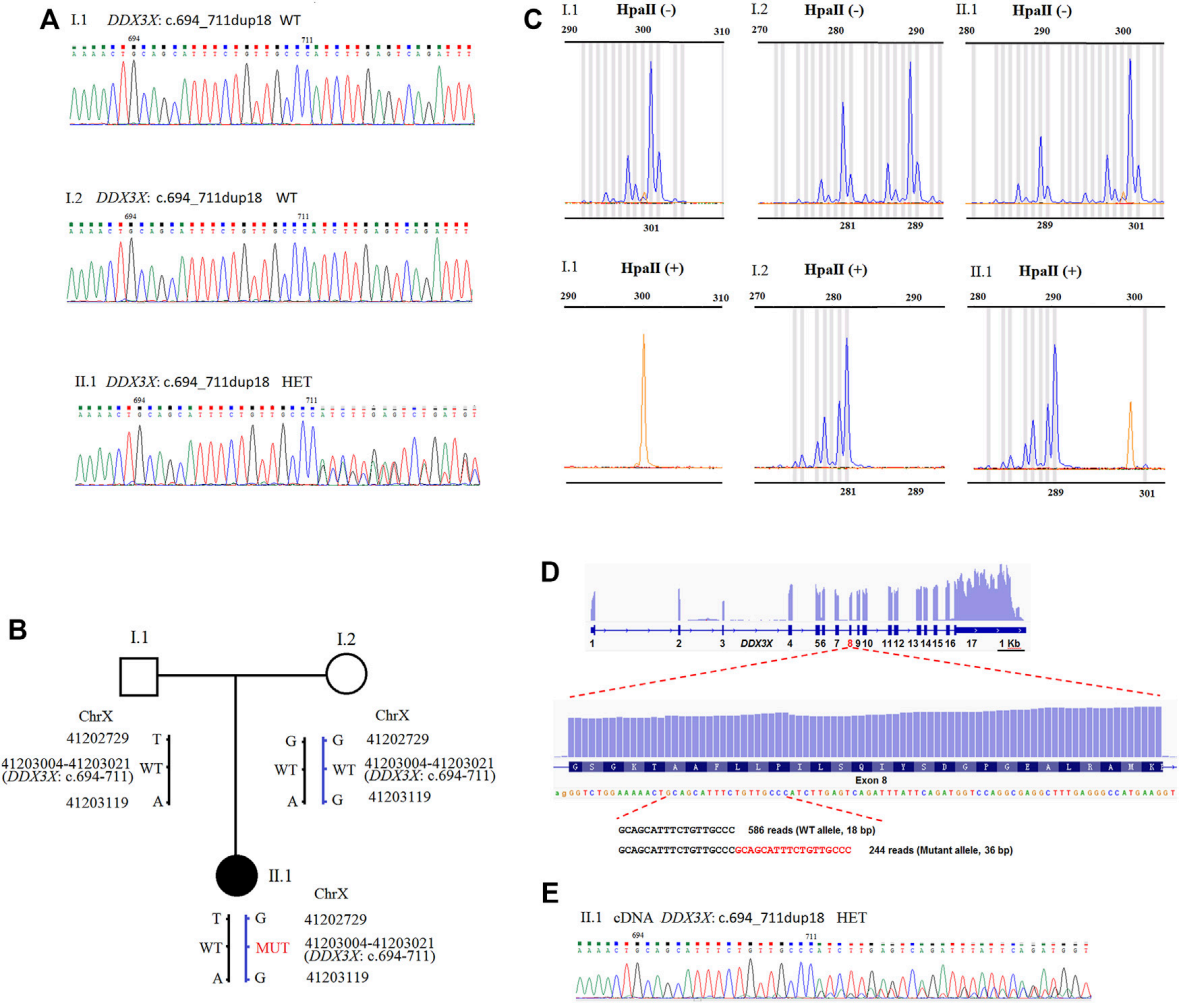
**(A)** Sequence analysis of genomic DNA from family members. The genotypes of *DDX3X* were wild type, wild type, and c.694_711dup18 HET, in I.1 (father), I.2 (mother), and II.1 (proband). **(B)** Schematic representation of the *DDX3X* mutation and two SNPs in the family. Besides the *DDX3X* c.694_711dup18, G > T and G > A variant were also detected at the positions NC_000023.10: g.41202729 and g.41203119 in II.1 by PCR-sanger sequencing. *DDX3X* c.694_711dup18 was confirmed to inherit from mother I.2 by the two SNPs. HET, heterozygote; WT, wild type; MUT, mutation type. **(C)** XCI pattern was detected by *AR* assay. A peak of 301 bp for *AR* was observed by assaying the undigested PCR product of I.1 and no peaks were observed for the HpaII digested product. The undigested PCR product of I.2 gave two peaks of 281 and 289 bp, and major peak of 281 bp was observed with the HpaII digested product. The undigested PCR product of the proband II.1 gave two peaks of 289 and 301 bp. One X-chromosome linked with the 289 bp peak of *AR* was inherited from the mother I.2 and the other from the father I1. The product of HpaII digestion gave only one major peak of 289 bp. II.1 exhibited extreme skewing of XCI. The inactivated X-chromosome was linked with the 289 bp peak of *AR*, which inherited from the mother I.2. **(D)** The IGV Genome Browser view showing RNA-seq reads mapped to *DDX3X* locus. The numbers represent exon number of *DDX3X* gene. Read number of wild type allele and mutant allele detected by RNA-seq is shown on the bottom. **(E)** Sequence analysis of the RT-PCR product from PMBCs of II.1. The mutant peaks were lower than the wild. HET, heterozygote.

Trio-based exome sequencing revealed a *de novo* heterozygous variant of NM_001193416.3: c.694_711dup18 (NC_000023.10: g.41203004_41203021dup18) in *DDX3X* gene of the female proband. Sanger sequencing verified the *de novo* variant in the proband, the parents were wild-type (Figure 1), both paternity and maternity were confirmed (PS2). This variant was absent from gnomAD, ESP and 1,000 Genomes databases (PM2). It had not been reported in the HGMD and ClinVar databases. It caused protein length changes as a result of six amino acids insertions (Ala232_Pro237dup) on the non-repeated region (PM4). Based on the American College of Medical Genetics (ACMG) guidelines (Richards et al., 2015), the variant was classified as likely pathogenic (1PS + 2PM).

Despite the fact that the variant was *de novo*, we were able to trace its origins back to the SNPs of PCR-sanger sequencing. Using a pair of PCR primers, we not only verified the variant, but also found two SNPs in the family. As presented in Supplementary Figure S1A, a heterozygous variant NC_000023.10: g.41202729G>T was detected in the proband II.1 by forward sequencing. As reverse sequencing showed that the base in this position was T, which was inherited from the father I.1. The in-frame duplication originated from the mother I.2. It indicated that the variant of NM_001193416.3: c.694_711dup18 occurred on the maternal X chromosome. The result was also confirmed by another SNP NC_000023.10: g.41203119G>A (Supplementary Figure S1B). The results were further confirmed by T-A cloning, sanger sequence chromatogram of single allele showed that the bases in two SNPs (NC_000023.10: g.41202729 and g.41203119) linked with the duplication were all G, which was inherited from the mother I.2. It revealed that the duplication existed on maternal allele (Supplementary Figure S2).

### *AR* assays showed that the proband possessed extremely skewed X inactivation, favoring the wild-type allele in peripheral blood

We examined XCI patterns using PCR-based *AR* (androgen receptor) assay in the family. After digestion with the methylation-sensitive restriction enzyme HpaII, only the allele on the inactive X chromosome could be amplified by PCR. Meanwhile, segregation analysis was used to determine the origin of the inactivated X chromosome. The undigested PCR product of II.1 yielded two peaks of 289 bp and 301 bp, respectively. For the HpaII-digested product, a major peak representing 289 bp was observed (Figure 1C), indicating the inactivated X chromosome inherited from the mother I.2. It was discovered that the X chromosome with the *DDX3X* variant of II.1 is inherited from the mother. It revealed that the X chromosome with the *DDX3X* variant from the mother had extreme skewing of XCI.

### Most X inactivation genes on the mutant X-chromosome of the proband have lower transcription by allele-specific expression analysis

To distinguish whole transcription on two X chromosomes of the proband II.1, we explored the exome sequencing data for heterozygous informative SNPs on the X chromosome of the proband to assess the parental origin. Combining RNA sequencing, informative SNPs were quantified allelic read counts. For the proband II.1, we found that most genes on the maternal X-chromosome were lower transcription, especially the XCI genes were almost no expression on the maternal X-chromosome (Supplementary Table S1). It was consistent with the result by *AR* assay. It revealed that the maternal mutant X chromosome was extremely inactivated in the proband.

### Lower expression of the allele with *DDX3X* mutation was detected by RNA sequencing, RT-PCR, and sanger sequence in peripheral blood mononuclear cells

In peripheral blood mononuclear cells (PMBCs), RNA sequencing revealed no differences in *DDX3X* total expressions between the proband II.1 and the mother I.2 (data not shown). We found *DDX3X* expressions in both wild and mutant alleles. The read counts for the *DDX3X* wild and mutant alleles were 586 and 244 (71:29), respectively, indicating that the mutant allele had lower expression (Figure 1D). We also identified the results using RT-PCR and Sanger sequence from PMBCs, and the mutant peaks were lower than the wild ones (Figure 1E). Meanwhile, it showed that the duplication and base G in NC_000023.10: g.41203119 were all in the same RNA-seq reads, further confirming the duplication located on maternal allele (Supplementary Figure S3).

## Discussion

*DDX3X* encodes a 662-amino-acid protein, its functional core contains highly conserved helicase core composed of two RecA-like domains (D1, D2) and N- and C-terminal extensions. As one of the most intolerant genes, tolerated variations in *DDX3X* are extremely rare (Snijders Blok et al., 2015). The mutation of *DDX3X*: c.694_711dup18 (p.Ala232_Pro237dup) locates on the communication between RNA and ATP binding sites of D1 domain. Three mutations (c.698C > T/p.A233V, c.704T > A/p.L235Q, c.704T > C/p.L235P) have been reported in the duplicated region, and A233V, as a recurrent mutation, was confirmed to impair helicase activity by functional studies (Snijders Blok et al., 2015; Turner et al., 2019; Lennox et al., 2020). In our study, *DD*X3X: c.694_711dup18 caused DDX3X protein length changes as a result of six amino acids insertions of the highly conserved region (Supplementary Figure S4), and altered protein topology (Supplementary Figure S5), which might affect its function.

*DDX3X* is one of the most common X-linked genes linked to intellectual disability ranging from mild to severe in females, but some females with *DDX3X* variants have no symptoms. Many studies have tried to explore genotype-phenotype correlations. Functional studies in zebrafish have shown that mutant *DDX3X* alleles, which found in male patients inherited from the normal mothers, had wild-type or near wild-type activity, suggesting that they are hypomorphic alleles, and both *DDX3X* missense and nonsense mutations function in a haploinsufficient manner (Snijders Blok et al., 2015; Kellaris et al., 2018).

In addition to loss of function (LoF), Lennox et al. (2020) recently elucidated that the same recurrent *de novo* variants (R326, I415, T532) had similar phenotypes, and missense variants were more likely to have abnormal brain structural, such as polymicrogyria (PMG). Clinical severity of *DDX3X*-related neurodevelopmental disorder was linked to reduced helicase activity and RNA-protein granules, missense variants could function in a dominant-negative manner (Lennox et al., 2020; Dai et al., 2022).

*DDX3X* is an XCI–escaping gene, the role of skewed XCI in phenotypic differences of *DDX3X*-related ID is unclear. Research in this field is limited. As Table 1, initially, Snijders Blok et al. (2015) reported that 6 out of 15 females with *DDX3X* mutations and ID had almost complete skewing of X-inactivation (>95%), which was higher than typical female populations, but it was unknown which X-chromosome was inactivated due to most mutations were *de novo*. Subsequently, Fieremans et al. (2016) reported two females with *DDX3X* mutations and ID were extreme skewing XCI, and only the reference allele was present at cDNA level in a *de novo* stop variant (NM001193417: p.G177X). At the transcriptional level, the mutant allele was not detected, but nonsense mediated mRNA decay could not be ruled out, so they could not conclude the mutant allele must be inactive. Meanwhile, the parental origin of the variant was not definite, so it could not distinguish which X-chromosome was inactivated by *AR* assay in DNA methylation level.

**TABLE 1:** Skewed XCI of female intellectual disability (ID) patients with variations in the escaping or variable XCI genes.

	Patient	Gene	XCI pattern	Variation	Inheritance	Variation on Xm or Xp	*AR* assay	RNA/cDNA assay (wt:mut)	Variation on Xa or Xi
This study	Case 1	*DDX3X*	Escaping	NM_001193416.3:c.694_711dup18: p.A232_P237dup	*De novo*	Xm	100:0	586:244	Xi
Miyake et al. (2013)	Case 2	*KDM6A*	Escaping	NM_021140.2: c.1909_1912delTCTA:p.Ser637Thrfs53	*De novo*	NA	98:2	wt:mut similar level	NA
Snijders Blok et al. (2015)	Case 3–8	*DDX3X*	Escaping	NA	NA	NA	>95:5	NA	NA
Fieremans et al. (2016)	Case 9	*DDX3X*	Escaping	NM_001193417.2: c.856G>A:p.G286S	Likely	NA	99:1	NA	NA
	Case 10	*DDX3X*	Escaping	NM_001193417.2: c.529G>T:p.G177X	*De novo*	NA	92:8	100:0	NA
	Case 11	*SMC1A*	Variable	NM_006303.3: c.2351T>C:p.I784T	*De novo*	NA	93:7	mut allele was lower	Xi
Vianna et al. (2020)	Case 12	*NLGN4X*	Escaping	NM_001282146.1: c.2284C>G:p.L762V	*De novo*	NA	98:2	NA	NA
	Case 13	*USP9X*	Escaping	NM_001039590.3: c.4290dupC:p.H1430fs	*De novo*	NA	97:3	100:0	NA

NA, unknown or undetected; Xm, maternal X chromosome; Xp, paternal X chromosome; wt, wild-type allele; mut, mutant allele; Xa, active X chromosome; Xi, inactive X chromosome.

In our study, we reported a *de novo DDX3X* mutation in a female with intellectual disability, but we were able to find the mutation occurred on the maternal X chromosome based on SNPs detected by PCR-sanger sequencing, and we confirmed that the mutant X chromosome was inactive detected by *AR* assay and RNA sequencing both in DNA methylation and transcriptional levels. Females with *DDX3X* mutations on the inactive X chromosome were still affected, according to the findings. Skewed XCI was insufficient to reverse the phenotype of XCI-escaping gene *DDX3X* deficiency.

**FIGURE 2:**
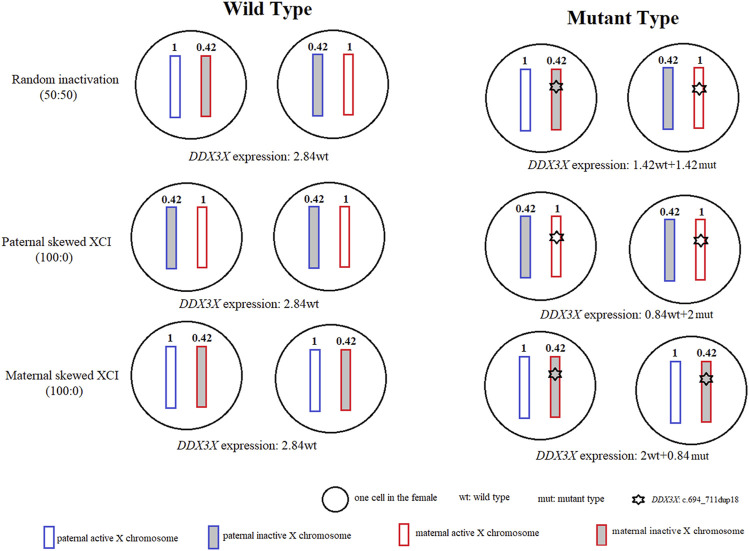
Modeled *DDX3X* expression levels in normal females and females with *DDX3X* mutation. For two normal female cells, there were no differences for *DDX3X* expressions in random XCI, paternal and maternal skewed XCI, all were 2.84. For two female cells with *DDX3X* mutation. The wild type of *DDX3X* expressions respectively were 1.42, 0.84, 2 in random XCI, paternal and maternal skewed XCI, which were affected by the XCI pattern. The white and gray boxes represented the active X chromosome (Xa) and inactive X chromosome (Xi). The blue and red showed the paternal and maternal X chromosome. The number inside the X chromosome indicated the proportion of *DDX3X* expression according to our data. wt, wild type; mut, mutation type.

As most XCI-escaping genes, including transcripts of *DDX3X*, are not fully expressed from the Xi, escaping ratios of *DDX3X* range from 29% to 61% (Garieri et al., 2018). Using RNA sequence, escaping ratio in our study is approximately 42% (244/586). If we only considered escaping XCI and skewed XCI, we would assume that *DDX3X* expression on the active X chromosome (Xa) was 1 in one cell, and according to our data, its expression on the inactive X chromosome (Xi) was 0.42 (1*42%). As shown in Figure 2, if we just calculated *DDX3X* expression from two cells. *DDX3X* expressions of wild type (wt) in the normal female with random XCI, paternal and maternal skewed XCI all were 2.84 (1 + 0.42 + 1 + 0.42), whereas *DDX3X* expressions of wild type and mutant type in maternal skewed XCI pattern of were 2 (1 + 1) and 0.84 (0.42 + 0.42). The proband with about 70% (2/2.84) normal and 30% (0.84/2.84) mutated *DDX3X* expression was still affected.

For the *DDX3X* in-frame insertion, we did not confirm if LoF or dominant-negative manner, play a key role in the pathogenic mechanism, and did not know the degree of LoF or dominant-negative caused by the mutation. If the mechanism was LoF, it indicated that *DDX3X* was very sensitive to gene dosage. If we assumed the function of mutated DDX3X completely lost. It showed that not only 50% normal DDX3X product by the single wild-type X chromosome was not sufficient for complete function, even though normal DDX3X dosage increased up to 70% by skewed XCI on the mutant X chromosome, the female with *DDX3X* heterozygous mutation still affected. In mice, Lennox et al. (2020) also reinforced the exquisite dosage sensitivity of the developing brain to *DDX3X*, as even a 25% reduction in DDX3X levels strongly perturbed neurogenesis.

In consideration of no differences in DDX3X total expressions between the proband II.1 and the mother I.2. This variant was more likely to behave like a missense than a loss-of-function, the pathogenic mechanism was more likely to dominant-negative manner. If we assumed all mutated DDX3X proteins were detrimental, it suggested that only 30% mutated *DDX3X* expression was enough to cause abnormal clinical phenotype, the expression of mutation did not have to come to 50%. No matter which mechanism acts, it does not change the conclusion, the female with *DDX3X* mutation in inactive X chromosome was still affected.

Other escaping or variable XCI genes associated with intellectual disability (ID) (Table 1), such as *SMC1A* and *USP9X*, are in a similar situation (Limongelli et al., 2010; Gervasini et al., 2013; Fieremans et al., 2016; Vianna et al., 2020). Variants in these genes were found to cause both X-linked ID and skewing of XCI, with the variant allele expressing less than the wild-type allele. However, the exact mechanisms underlying XCI skewing in females harboring variants in escape XCI genes remain unknown. Perhaps these genes were X-Y homologous genes, which were too important for life, as females with mutations on Xa were difficult to survive.

Of course, there are some limitations in our study. Most *DDX3X* variants are *de novo*, tracing their origins of parents are usually ignored, and then it is unable to assess the XCI status on the methylation level. Our research is just a case report, providing a new idea to detect XCI status, further studies in a larger cohort with different variation types are needed. In addition, we just detected the XCI status of blood, the status of brain was unknown. Although Werner et al. (2022) recently modeled variability in human XCI ratios across tissues and individuals using the GTEx dataset, determining that XCI ratios are consistent across all tissue lineages. Due to no sufficient evidence, we could not confirm that XCI status must be consistent between blood and brain in patients with *DDX3X* variants. It remains to be proved by further experiments.

In conclusion, we described a *de novo DDX3X* mutation in a female with intellectual disability. Using the *AR* assay and RNA sequencing, we verified that the X chromosome with the *DDX3X* mutation was extreme skewing inactivation. However, it was insufficient to reverse the phenotype of *DDX3X*-related neurodevelopmental disorder. It contributed to our understanding of the role of skewed XCI in the phenotypic differences, which can aid in the genetic counseling and prenatal diagnosis of disorders in females with *DDX3X* defect.

## Data Availability

The datasets for this article are not publicly available due to concerns regarding participant/patient anonymity. Requests to access the datasets should be directed to the corresponding author.
