# Supplementary figures and images for: Case Report: De novo DDX3X mutation caused intellectual disability in a female with skewed X-chromosome inactivation on the mutant allele
Source: Front Genet. 2022 Oct 10;13:999442. doi: 10.3389/fgene.2022.999442 (PMC9589230; doi:10.3389/fgene.2022.999442)

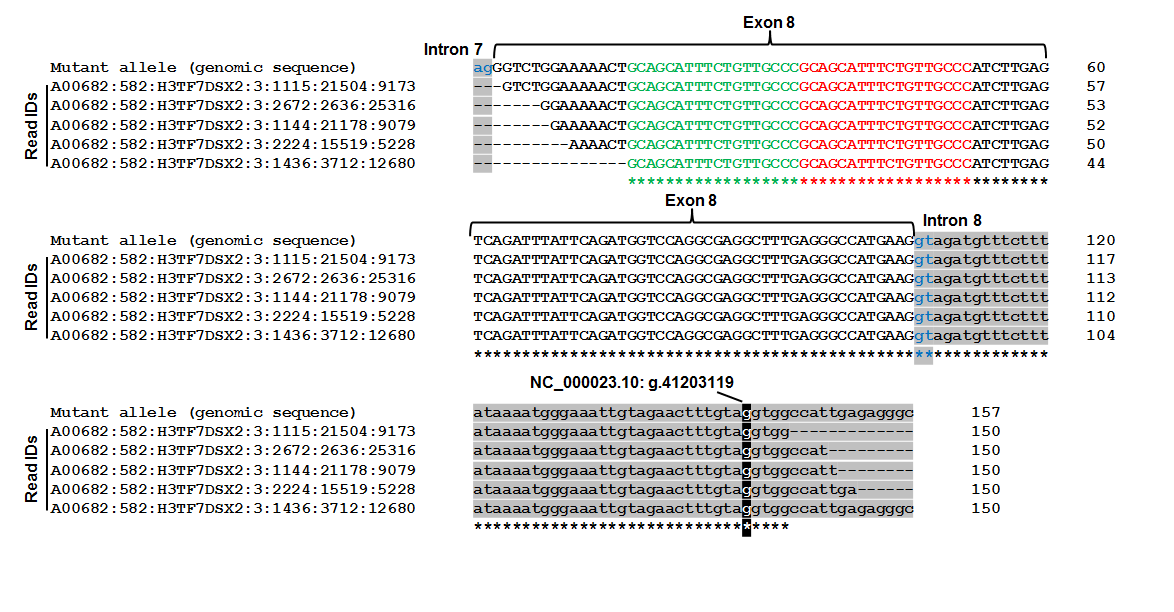

Supplement: Supplementary file 1 [file Image3.TIF]

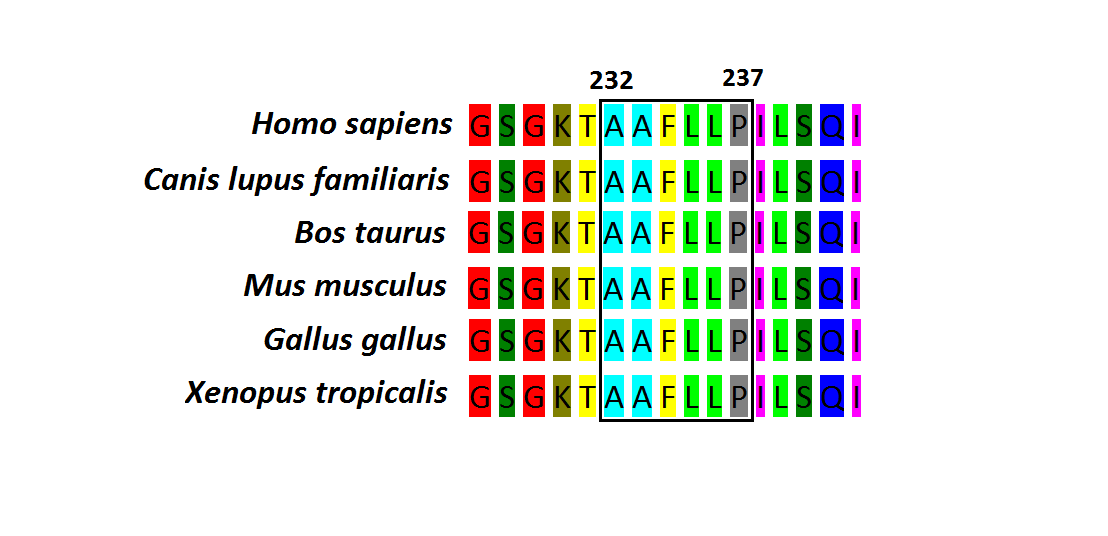

Supplement: Supplementary file 2 [file Image4.TIF]

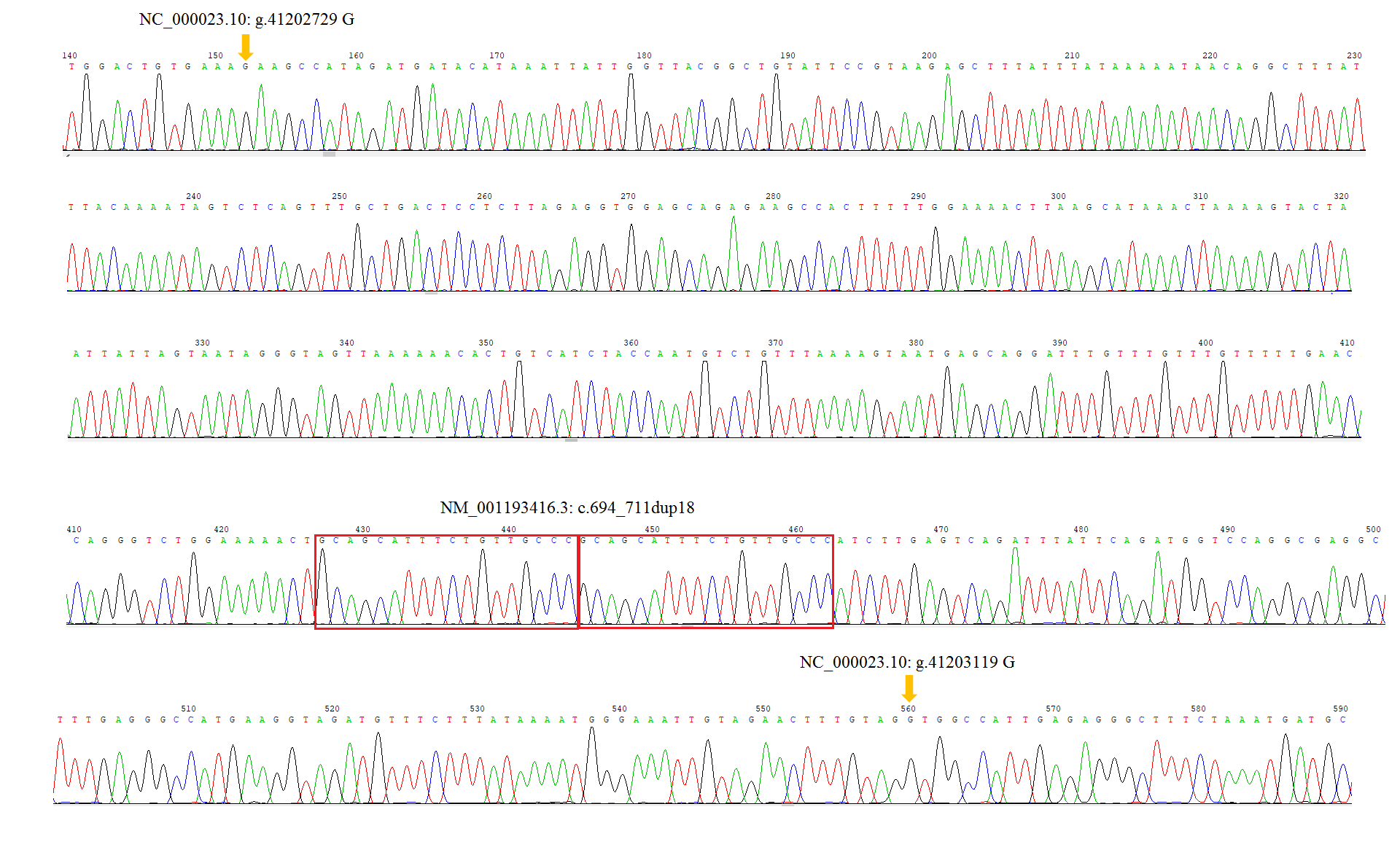

Supplement: Supplementary file 3 [file Image2.TIF]

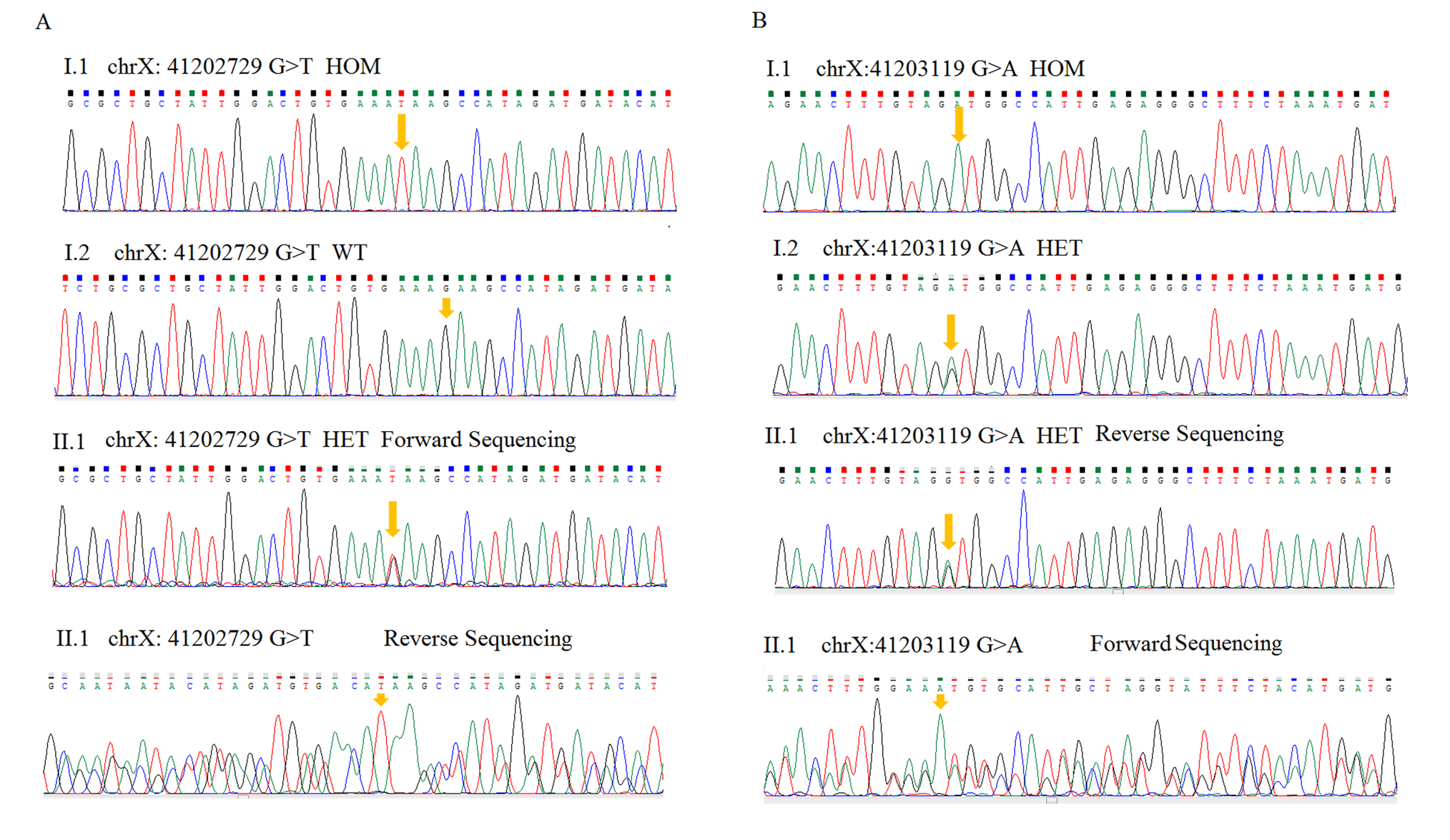

Supplement: Supplementary file 4 [file Image1.TIF]

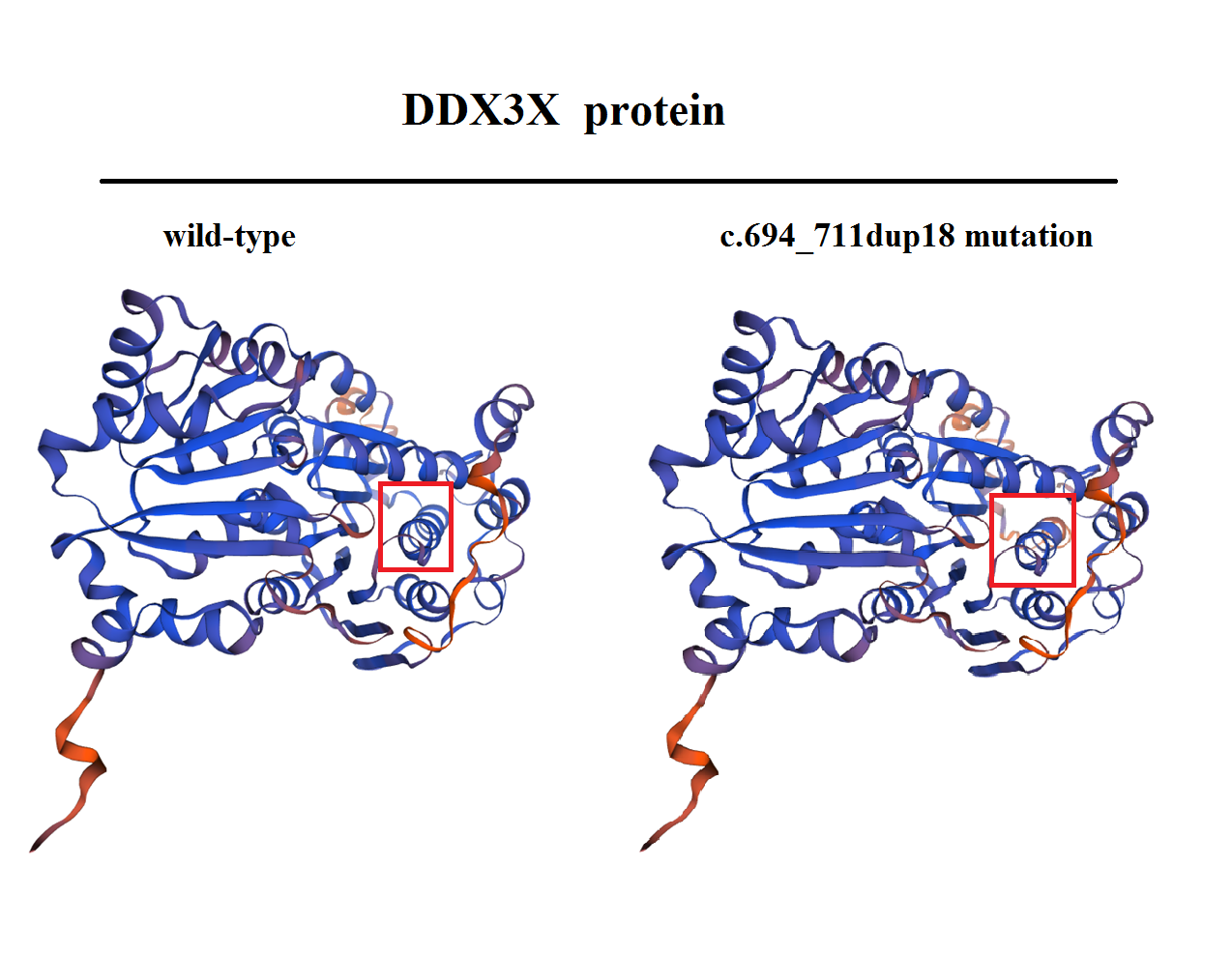

Supplement: Supplementary file 7 [file Image5.TIF]
