# Supplementary material for: Case Report: De novo DDX3X mutation caused intellectual disability in a female with skewed X-chromosome inactivation on the mutant allele
Source: Front Genet. 2022 Oct 10;13:999442. doi: 10.3389/fgene.2022.999442 (PMC9589230; doi:10.3389/fgene.2022.999442)
Supplement: Supplementary file 5 [file Table2.DOCX]

**Legends**

**Figure S1.** Sequence analysis of two SNPs at the positions NC_000023.10: g.41202729 and g.41203119 from family members. (A) The genotypes of NC_000023.10: g.41202729 were C>T HOM, wild type, and C>T HET, in I.1 (father), I.2 (mother), and II.1 (proband) by forward sequencing. As reverse sequencing showed that the base in this position was T, which inherited from the father I.1. The frameshift allele originated from the mother I.2. (B) The genotypes of NC_000023.10: g.41203119 were G>A HOM, G>A HET in I.1 (father), I.2 (mother) by forward sequence, and G>A HET in II.1 (proband) by reverse sequencing. As forward sequencing showed that the base in this position was A, which inherited from the father I.1. The frameshift allele originated from the mother I.2. The results indicated that the variant of NM_001193416.3: c.694_711dup18 occurred on the maternal X chromosome. HOM: homozygote; HET: heterozygote; WT: wild type; MUT: mutant type.

**Figure S2.** Sanger sequence chromatogram of single allele after T-A cloning. The bases in two SNPs (NC_000023.10: g.41202729 and g.41203119) linked with the duplication (NM_001193416.3: c.694_711dup18) were all G, which was inherited from the mother I.2.

**Figure S3.** Multiple alignment showing RNA-seq reads mapped to exon 8 and adjacent introns (grey) of *DDX3X*. Green and red represent WT (18 bp) and mutant alleles (36 bp). Blue represents acceptor site and donor site of intron 7 and intron 8, respectively. It shows that the duplication and base G in NC_000023.10: g.41203119 are all in the same reads, further confirming the duplication locates on maternal allele.

**Figure S4.** Alignment of multiple DDX3X protein sequences across species. The *DD*X3X: c.694_711dup18 resulted in 6 amino acids of DDX3X protein (residues 232–237) duplicated in the highly conserved amino acid region in different species. The black column shows the duplicated amino acids.

**Figure S5.** The structures of wild-type and c.694_711dup18 mutation DDX3X protein as predicted by the software SWISS-MODEL (https://swissmodel.expasy.org/).
